# Divergent Roles of Mitochondria Dynamics in Pancreatic Ductal Adenocarcinoma

**DOI:** 10.3390/cancers14092155

**Published:** 2022-04-26

**Authors:** Cristian Andres Carmona-Carmona, Elisa Dalla Pozza, Giulia Ambrosini, Andrea Errico, Ilaria Dando

**Affiliations:** Department of Neurosciences, Biomedicine and Movement Sciences, University of Verona, 37134 Verona, Italy; elisa.dallapozza@univr.it (E.D.P.); giulia.ambrosini@univr.it (G.A.); andrea.errico@univr.it (A.E.)

**Keywords:** mitochondrial dynamics, PDAC, cancer stem cells, metabolism, molecular target

## Abstract

**Simple Summary:**

Pancreatic ductal adenocarcinoma is one of the most lethal neoplasia due to the lack of early diagnostic markers and effective therapies. The study of metabolic alterations of PDAC is of crucial importance since it would open the way to the discovery of new potential therapies. Mitochondria represent key organelles that regulate energy metabolism, and they remodel their structure by undergoing modifications by fusing with other mitochondria or dividing to generate smaller ones. The alterations of mitochondria arrangement may influence the metabolism of PDAC cells, thus supporting the proliferative needs of cancer. Shedding light on this topic regarding cancer and, more specifically, PDAC may help identify new potential strategies that hit cancer cells at their “core,” i.e., mitochondria.

**Abstract:**

Pancreatic ductal adenocarcinoma (PDAC) is one of the most aggressive tumors; it is often diagnosed at an advanced stage and is hardly treatable. These issues are strictly linked to the absence of early diagnostic markers and the low efficacy of treatment approaches. Recently, the study of the metabolic alterations in cancer cells has opened the way to important findings that can be exploited to generate new potential therapies. Within this scenario, mitochondria represent important organelles within which many essential functions are necessary for cell survival, including some key reactions involved in energy metabolism. These organelles remodel their shape by dividing or fusing themselves in response to cellular needs or stimuli. Interestingly, many authors have shown that mitochondrial dynamic equilibrium is altered in many different tumor types. However, up to now, it is not clear whether PDAC cells preferentially take advantage of fusion or fission processes since some studies reported a wide range of different results. This review described the role of both mitochondria arrangement processes, i.e., fusion and fission events, in PDAC, showing that a preference for mitochondria fragmentation could sustain tumor needs. In addition, we also highlight the importance of considering the metabolic arrangement and mitochondria assessment of cancer stem cells, which represent the most aggressive tumor cell type that has been shown to have distinctive metabolic features to that of differentiated tumor cells.

## 1. Pancreatic Cancer and Mitochondria Alterations

### 1.1. Pancreatic Cancer

Pancreatic cancer (PC) is one of the most aggressive neoplasia’s associated with an awful prognosis and a 5-year survival rate of around 10% [1]. Genetic events represent a class of risk factors: inherited genetic changes in 5–10% of cases may be exploited to characterize populations who benefit from prevention or prompt early detection [2]. Due to the location of the organ, pancreatic cancer may be difficult to detect and is often diagnosed in an advanced stage of the disease [1]. The most frequent type of pancreatic cancer is ductal adenocarcinoma (PDAC), which accounts for more than 90% of cases and is developed in the exocrine compartment. At present, the best way to detect PDAC is a combination of medical imaging techniques, such as ultrasound or computed tomography, blood tests, and examination of tissue samples (biopsy) [3]. However, patients typically present an advanced disease due to the lack of symptoms or to the presence of symptomatic events not attributable to cancer when it is already localized. Almost 60–70% of PDAC cases arise from the head of the pancreas and are usually diagnosed earlier than tumors arising from the body and tail since the head of the pancreas contains the common bile duct [4]. Patients with pancreatic cancer are often divided into one of four categories based on the extent of the disease: resectable, borderline resectable, locally advanced, and metastatic. In addition, the evaluation of patient condition is an important factor considered during diagnosis [1]. Surgical resection represents the only chance for cure. Still, the overall survival for metastatic pancreatic cancer remains poor, with less than 20% of patients that survive after the first year from the diagnosis. Surgical resection and chemotherapy (generally represented by gemcitabine/nab-paclitaxel or FOLFIRINOX, a combination of oxaliplatin, irinotecan, fluorouracil, and leucovorin) have managed to improve the survival of patients with early-stage pancreatic cancer. Still, these treatments are not sufficient for patients with late stages of the disease [5].

In recent years, growing evidence has shown that metabolism plays a critical role in cancer carcinogenesis and progression. In particular, PDAC cells present alterations of different metabolic pathways in comparison to normal cells in the same tissue. Indeed, pancreatic cancer cells alter key pathways, including glycolysis, oxidative phosphorylation, amino acid, and lipid metabolism, to adapt and sustain their energetic requirements for uncontrolled proliferation, also under the influence of the tumor microenvironment [6]. Since mitochondria represent the “core” of energy production and mitochondrial dynamics are strictly linked to metabolic reprogramming, in this review, we described the regulation of their morphology and function in pancreatic cancer cells, opening the way to the identification of new potential biomarkers and therapeutic targets based on mitochondria, aspiring in the improvement of diagnosis/treatment efficacy.

### 1.2. Pancreatic Cancer Cells Show Mitochondrial and Metabolic Defects

Overall, mitochondria have diverse functions in the regulation of cell signaling and biochemical pathways. Thus, their alterations are linked with many diseases, and cancer is not an exception. In fact, the connection between mitochondrial function and metabolism regulation with cancer development has been reviewed by different groups [7,8,9,10].

One of the most important metabolic hallmarks of cancer cells is the upregulation of glycolysis even in the presence of oxygen, known as the Warburg effect [11,12]. The “aerobic glycolysis” provides several intermediates needed to synthesize macromolecules required for rapid proliferation. It also contributes to maintaining redox balance and enhances cell cancer invasion [13]. Initially, it was assumed that glycolysis upregulation was a consequence of mitochondrial impairment. However, this vision has been re-valuated based on further studies demonstrating that tumor mitochondria support respiration and ATP production [14,15,16]. The idea that cancer cells switch from oxidative phosphorylation to glycolysis has also been challenged by the evidence that cancer cells show distinct metabolic requirements determined by genetic background and tumor microenvironment [17]. In the case of pancreatic adenocarcinoma, even in the same patient, primary tumor and metastatic lesions may exhibit different genic expression and metabolic alterations [18,19]. In pancreatic cancer cells, diverse genetic alterations are considered responsible for metabolic rewiring, especially in modulating mitochondrial function to support carcinogenesis. Among the most relevant of these alterations are the oncogenic *KRAS* mutations, which occur in more than 90% of PDAC cases [20]. These mutations render KRAS constitutively active, promoting tumor growth and evasion of immune destruction. Cells carrying *KRAS* mutations are characterized by increased glucose uptake and flux through glycolysis due to KRAS-dependent regulation of glycolytic enzyme expression. This glycolytic phenotype correlates with poorer prognoses in patients with PDAC [20]. Furthermore, KRAS-driven cancer cells modulate the total mitochondrial content by inducing mitophagy. In this process, cells recycle tricarboxylic acid (TCA) cycle metabolites required for biosynthesis and bioenergetics pathways [21]. Finally, KRAS signaling has an important role in maintaining the ideal balance of mitochondrial fission and mitochondrial fusion, the process known as mitochondrial dynamics.

## 2. Mitochondria Dynamics

Mitochondria are not rigid structures; instead, they have a high degree of shape modifications, and their subcellular distribution is always changing according to cell requirements. Indeed, mitochondria can combine, forming networks (fusion), whereas mitochondria can divide into small fragments (fission) in other conditions. In terms of energy demand, fragmented mitochondria are associated with a rich nutrient environment, whereas elongated mitochondria are related to starvation [22]. In fact, mitochondrial elongation leads to an increase in energy efficiency to maintain ATP production when the availability of nutrients is limited [23]. In addition to content mixing, mitochondrial dynamics allow the selective removal of dysfunctional mitochondria, ensuring a healthy population of mitochondria in the cell, mainly through mitophagy. The balance between fusion and fission is involved in cell death, calcium homeostasis, cell respiration, and autophagy. The alterations in mitochondrial dynamics have been associated mainly with neuropathies and neurodegenerative diseases [24]; however, the study of mitochondrial dynamics in metabolic diseases and cancer has increased considerably during the last decade, attributing a potential role in the origin and progression of these pathologies [25]. A deeper investigation of the molecular mechanisms underlying the alterations in the balance of fission/fusion might contribute to the identification of potential therapeutic targets for the development of new treatments for cancer. Previous reviews provided detailed information about mitochondrial dynamics [25,26,27]; here, we describe the main players in this process to improve our understanding of how fusion/fission can impinge on pancreatic cancer progression.

### 2.1. Mitochondrial Fusion

Mitochondrial fusion is the binding of two mitochondria into one larger. This merge implies that both the outer membrane and inner membrane fuse while the matrix components mix to form a new mitochondrion. This process requires mainly three GTP-hydrolyzing enzymes of the dynamin family: the mitofusins, MFN1 and MFN2 are required for outer mitochondrial membrane (OMM) fusion, whereas OPA1 is involved in inner mitochondrial membrane (IMM) fusion. The function of both mitofusins is essential for embryonic development because mice deficient in either MFN1 or MFN2 die in midgestation [28]. Nevertheless, the overexpression of MFN1 or MFN2 in fibroblasts deficient in MFN2 or MFN1, respectively, can restore the mitochondrial network, proving that these proteins have redundant functions to promote mitochondrial fusion [28]. The expression of mitofusins is regulated by transcription factors, including PGC1β [29], involved in mitochondrial biogenesis and oxidative phosphorylation. In addition to its function as a mitochondrial fusion protein, the importance of MFN2 in oxygen consumption and the activity of electron transport chain (ETC) complexes have been widely documented in muscle and liver cells [30,31,32]. However, it has been reported that cells completely deficient in the *MFN2* gene can develop adaptive mechanisms that maintain the oxidative phosphorylation (OXPHOS) function in the long term, showing that MFN1 might compensate for the loss of MFN2 to maintain energy metabolism [33]. In addition, a double knockout of *MFN1* and *MFN2* in fibroblasts showed a severely reduced oxygen consumption rate and OXPHOS protein content, sustaining the important role of these two proteins in support of energy metabolism [33]. Moreover, MFN2 is also expressed in the endoplasmic reticulum (ER) and modulates the interaction between ER and mitochondria, a phenomenon required for efficient mitochondrial Ca^2+^ uptake [34].

On the other hand, Optic Atrophy 1 (OPA1), which mediates the fusion of the inner mitochondrial membrane, has eight variants in humans, resulting from differential splicing of exons 4, 4b, and 5b [35]. The protein structure that includes S1 and S2 proteolysis sites is encoded by exon 5 and 5b, respectively. In addition, OPA1 has a mitochondrial-targeting sequence (MTS) that determines its location in the inner membrane. During import into the mitochondrial matrix, the MTS removal produces the long (L) isoforms, which are anchored to the inner membrane, with most of the protein facing the intermembrane space (IS). These L-isoforms are processed by OMA1 and YME1L in the S1 and S2, respectively, to generate short isoforms. These short isoforms do not have an anchor domain and could be soluble in the IS, but they are bound to long isoforms to modulate mitochondrial fusion [36,37]. The study of the individual OPA1 isoforms shows that, in general, long isoforms are more related to maintaining mitochondrial fusion, whereas short isoforms are more essential for energetic efficiency [38]. Interestingly, to completely restore mitochondrial network morphology, the balance of both long and short isoforms is necessary [39,40]. In addition, it has been shown that OPA1 works in concert with other proteins, including MICOS, YME1L, and Sam50, in the control of mitochondria cristae structure and dynamics. Indeed, OPA1 sustains the narrowing of cristae junction (CJ) and dimerization of complex V, an event that controls cristae biogenesis and morphology by inducing a positive curvature of the cristae tip. In a recent study, it has been demonstrated that the knockout of OPA1 decreases the fusion of mitochondria and alters the shape, length, and dynamics of cristae, showing a marked decrease in cristae elongation and cristae-cristae contact [41].

### 2.2. Mitochondrial Fission

Mitochondrial fission or mitochondrial division is mediated mainly by DRP1, which is another large dynamin-related GTPase protein. This protein translocates from the cytosol into the outer mitochondrial membrane, where it binds its receptors (FIS1, MiD49, MiD51, and MFF) to initiate the mitochondrial constriction and, as a result, the division of a mitochondrion into two smaller ones [42]. The function of DRP1 is tightly regulated by post-translational modifications, where the phosphorylation mechanism is the most studied. Indeed, it has been demonstrated that phosphorylation at Ser616 by ERK2 stimulates mitochondrial fission [43], whereas the phosphorylation at Ser637 by PKA decreases DRP1 GTPase activity and leads to mitochondrial elongation [44]. In contrast, the role of DRP1 outer membrane receptors in the mitochondrial division is less clear. A conditional knockout model of FIS1 in colon carcinoma cells showed that FIS1 is dispensable for mitochondrial fission [45]. However, it can act in sequence with MFF at the ER-mitochondrial sites and favors some types of mitophagy [46]. Either MiD49 or MiD51 can mediate DRP1 recruitment in the absence of MFF or FIS1 [47]. Still, it has been shown that their overexpression increases the elongation of mitochondria due to sequestration and inactivation of DRP1 on the mitochondrial outer membrane, blocking fission [48]. Altogether, these studies demonstrate that multiple proteins can bind and recruit DRP1, thus affecting the mediation of mitochondrial division.

### 2.3. Physiological Significance of Fusion/Fission Balance

Different studies have widely demonstrated the importance of the maintenance of fusion/fission balance for mitochondrial function, especially through knockout experiments. As mentioned above, the ablation of either process in mice is embryonically lethal, while in cells, the silencing of the main proteins involved in fusion/fission results in severe cellular and mitochondrial dysfunction [28,49,50], including alterations in the cell cycle [51], apoptosis [52,53,54,55,56], self-renewal capacity [57,58], and reactive oxygen species (ROS) [59,60]. For instance, in cardiomyocytes, it has been shown that ROS induces mitochondrial fission by reducing the phosphorylation of DRP1 at Ser637 and by inhibiting the proteolytic processing of OPA1 [61].

The overall analysis of all these studies highlights that the function of mitochondrial dynamics largely depends on cell types, and generalizations should be avoided. Unfortunately, no studies in conditional KO mice reveal the role of fusion/fission in pancreas physiology, especially in acinar and ductal cells, which are the initiating cells for precursor lesions of pancreatic cancer. In the late 1990s, Petersen et al. found that living pancreatic acinar cells have the highest density of mitochondria between the apical granular region and the basal membrane of the cells, regulating cellular Ca^2+^ homeostasis [62,63]. However, mitochondrial dynamics were not evaluated. The investigation of mitochondrial dynamics in the pancreas has been focused extensively on β-cells and insulin secretion in the context of diabetes. In primary mouse β-cells, mitochondria are organized densely, forming tubular networks throughout the cytoplasm [64]. Interestingly, the induction of mitochondrial fragmentation by overexpression of FIS1 impaired glucose-stimulated insulin secretion [65]. This is a clear example of how an alteration of fission/fusion balance affects cell function and highlights the importance of studying normal cells’ physiological events. Therefore, more studies are necessary to characterize the normal role of mitochondrial dynamics in both ductal and acinar cells to understand the role of this process in the origin and progression of pancreatic cancer.

### 2.4. Mitochondria Dynamics and Cancer

Tumor cells are characterized by different alterations at the cellular and molecular level, which were compiled by Hanahan and Weinberg in 2011 as hallmarks of cancer and were recently updated [11,66]. As mentioned above, mitochondrial dynamics are involved in all these mechanisms, and the disruptions in mitochondrial fusion/fission balance have been reported in almost all types of cancer [25,67].

**Figure 1:**
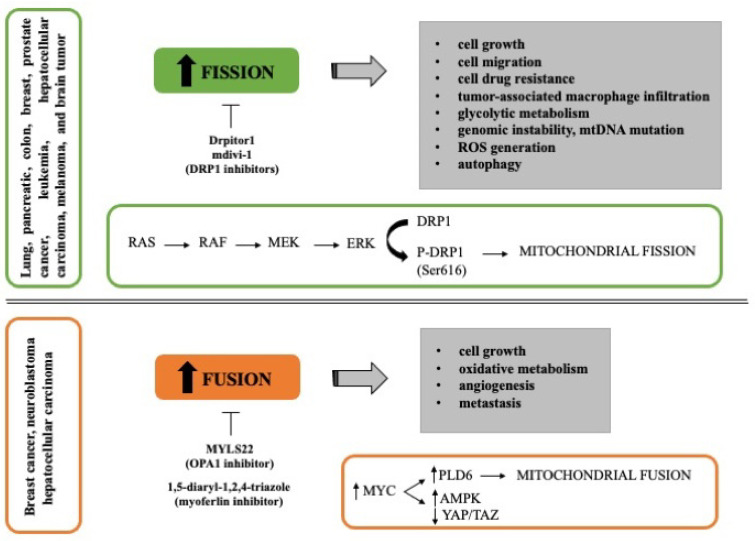
Mitochondrial dynamics in cancer. The figure schematically reports the role of mitochondria fission and fusion in different tumor types and the effect of inhibitors that can be potentially used in the clinic.

Many authors have found that mitochondrial fission drives malignant phenotype in different types of cancer, including pancreatic, colon, breast, prostate cancer, leukemia, hepatocellular carcinoma, melanoma, and brain tumor. It has been reported that mitochondrial fission promotes cell migration, tumor-associated macrophage infiltration, autophagy, and cell drug resistance [68,69,70]. Serasinghe et al. reported that the maintenance of fragmented mitochondria in cancer cells involved the MAPK pathway (RAS-RAF-MEK-ERK) since the inhibition of oncogenic MAPK signaling reduced DRP1 levels resulting in mitochondrial hyper-fusion and increased mitochondrial metabolism (Figure 1). In fact, DRP1 phosphorylation at Ser616 by ERK2 is enough to resemble neoplastic transformation-induced mitochondrial dysfunction [71]. In addition, RAS transformed cells present a constitutive mitochondrial fragmentation attributed to the activation of ERK2 [43]. Considering the common feature of mitochondrial fission in cancer, numerous authors have studied the inhibition of proteins involved in this process, especially DRP1. The silencing of DRP1 reduced the migration potential and increased genomic instability, generation of ROS, and mtDNA mutations in cancer cell lines [72]. Furthermore, the use of DRP1 inhibitors, such as mdivi-1 and Drpitor1, impairs oxidative metabolism and induces cell death [73,74], suggesting that inhibition of mitochondrial fission could be a prospective strategy to reduce tumor growth and increase the chemotherapy sensitivity (Figure 1).

In line with the above-reported studies, it has been shown that the activation of mitochondrial fusion correlates with improved survival in preclinical models of pancreatic cancer [75]. However, recently some studies suggest that mitochondrial fusion could support tumor cell growth in gynecologic cancer and liver tumor [76,77]. Both non-transformed and transformed mouse fibroblasts expressing *RAS* oncogene showed an increase in oxidative phosphorylation during proliferation supported by mitochondrial fusion; in fact, the deletion of *MFN2* was sufficient to reduce the cell proliferation [78]. Another study showed that tumor tissues and in vitro cultured tumor organoids from hepatocellular carcinoma patients have an excessive activation of mitochondrial fusion. In this case, the knockdown of *OPA1* or *MFN1* inhibited tumor formation in vivo in mice [77]. Furthermore, deletion of endothelial *OPA1* decreases tumor angiogenesis, growth, and metastasis in vivo models of melanoma and breast cancer, suggesting a possible role of OPA1 in the regulation of Ca^2+^ levels, NFκB signaling, and angiogenic gene expression [79]. The induction of mitochondrial fusion is in part mediated by *MYC* oncogene through the activation of PLD6, a phospholipase present in the mitochondrial outer membrane, which in turn increases the AMPK activity and decreases the YAP/TAZ pathway, maintaining the clonogenic activity of breast cancer cells (Figure 1) [68]. Considering that MYC is highly expressed in some cancers, such as breast cancer [68] and neuroblastoma [80], the search for inhibitors of mitochondrial fusion opens a new approach for these types of cancer as MYLS22 (OPA1 inhibitor) recently reported by Scorrano’s group [79].

Thus far, it is evident that mitochondrial dynamics are complex processes regulated by different molecules and microenvironmental conditions. Furthermore, distinct studies showed that the fusion/fission balance might be different according to the tumor cell type (Table 1).

Hence it is important to study this process by performing functional assays in each cancer type to identify new potential diagnostic markers and therapeutic targets. Below we discuss specifically the role of mitochondrial dynamics in pancreatic cancer, focusing on understanding how these dynamics are linked to tumor progression and chemoresistance.

## 3. Mitochondria Dynamics in Pancreatic Cancer

### 3.1. Mitochondrial Fusion in Pancreatic Cancer

Although PDAC cells generally exhibit abnormally fragmented mitochondria due in part to the expression of the oncogene *KRAS*, as described above, the role of mitochondrial fusion in this tumor remains controversial. Indeed, since mitochondrial fusion is more commonly observed in normal tissue in comparison to tumoral ones, some authors have hypothesized that the induction of this process could balance the mitochondrial dynamics and reduce oncogenicity [71,75]. In this regard, Yu et al. showed that the overexpression of MFN2 in both in vitro and in vivo models of PDAC promoted autophagy and a reduction in mitochondrial mass, oxygen consumption rate (OCR), and ATP production. This reduction in oxidative phosphorylation by MFN2 overexpression correlated with decreased cell proliferation increased G1 arrest and reduced metastatic lung colonization after tail vein injection. The pharmacological induction of mitochondrial fusion by leflunomide, the FDA-approved anti-arthritis drug, showed similar effects to MFN2 overexpression, improving survival in different mouse models of pancreatic cancer [75]. In addition to this observation, other authors demonstrated that the expression of MFN2 was significantly decreased in tumor tissues. At the same time, MFN2 overexpression affects the levels of proliferating cell nuclear antigen (PCNA) and endothelial growth factor A (VEGFA) in HUVEC cells, thus inhibiting cell growth and angiogenesis [89]. Furthermore, it has also been shown that the expression of MFN2 suppressed cell proliferation and induced cell autophagy in PDAC cells by inhibiting the PI3K/AKT/mTOR signaling pathway [90]. Altogether, these observations support the notion of exploiting mitochondrial fusion as an effective strategy in PDAC therapy. However, this approach could be a double-edged sword. Indeed, it is necessary to understand, for instance, the molecular mechanisms through which the induction of MFN2 reduces the OXPHOS metabolism in PDAC since numerous authors have reported that mitochondrial fusion favors an increase in energetic efficiency of both tumor and normal cells [30,91,92,93].

**Figure 2:**
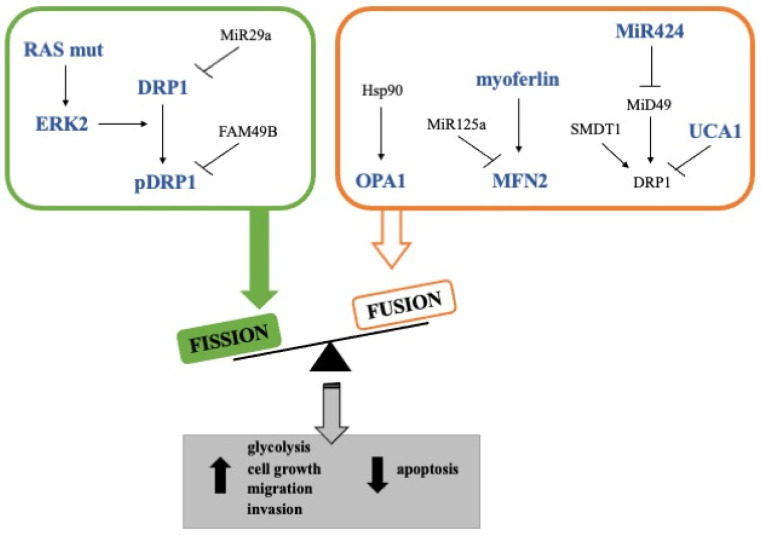
Mitochondrial dynamics in pancreatic cancer. The figure describes the molecular mechanisms involved in mitochondrial fission (green) and fusion (orange). Overexpressed proteins/miRNAs are indicated in blue.

Another protein that has been shown to support mitochondrial fusion in PDAC cells is FAM49B (family with sequence similarity 49-member B) (Figure 2) [94]. Indeed, it has been demonstrated that the loss of FAM49B in PDAC cells led to more punctuated mitochondria, decreased mitochondrial membrane potential, increased ROS generation, and induction of DRP1 phosphorylation at Ser616, supporting the notion that FAM41B absence favors mitochondrial fission. In addition, the silencing of FAM49B also resulted in enhanced PDAC cell proliferation and invasion, suggesting that this protein could act as a tumor suppressor by regulating mitochondrial dynamics and redox reactions [94]. However, it is noteworthy that the expression of this protein is higher in PDAC cell lines than in tumor tissues or cancer cells cultured in 3D, opening the possibility of a FAM49B downregulation in vivo by the tumor microenvironment [94].

Oppositely, although the inhibition of mitochondrial fusion in PDAC cells has not been studied in-depth, some other studies showed that some proteins involved in mitochondrial fusion are emerging as key molecules for cancer progression and chemoresistance. Among these, OPA1 has been identified as a prognosis-related gene in several cancers, and its expression at high levels has been found to correlate with a worse prognosis in PDAC patients [84,95]. In addition, the inhibition of the interaction between Hsp90 and OPA1 in PDAC tumors studied in KPC mice led to a reduction in mitochondrial cristae amount and energy production [96]. Moreover, the proteolytic activity of YME1L, which is strictly linked with OPA1 and is regulated post-translationally by mTORC1, has been shown to be required for PDAC cell growth in culture systems or xenografted nude mice [97,98]. Although this evidence suggests a pro-tumorigenic role of mitochondrial fusion, other studies should be carried out to determine if the pharmacological inhibition of OPA1 in PDAC cells curtails tumor growth by angiogenesis inhibition, as has already been reported in vivo models of melanoma and breast adenocarcinoma [79,84], or directly affects tumor cells. Furthermore, it has been shown that the knockdown of UCA1, an lnc-RNA upregulated in PDAC patients, increases DRP1 expression and phosphorylation, thus further supporting the role of mitochondria fusion in the sustainment of cancer proliferation (Figure 2) [83]. In line with this, Rademaker et al. found that the loss of myoferlin, a protein involved in mitochondrial fusion and overexpressed in PDAC, induced mitochondrial fission, decreased ATP production, and reduced cell proliferation, induction of autophagy but not apoptosis (Figure 2). Analysis with 18F-deoxyglucose positron emission tomography showed that myoferlin level correlated with the tumor size and glycolytic activity. In addition, PDAC patients with a high myoferlin expression have significantly shorter survival than patients with low myoferlin expression [81]. Accordingly, in a murine in vivo model, myoferlin was significantly involved in cell migration and metastatic capacity of PDAC cells [99]. To better delineate this scenario, it is noteworthy that the mechanism underlying the role of myoferlin in mitochondrial structure implies the interaction of this protein with mitofusins. Indeed, it seems that myoferlin takes part in the sequestration of MFN2, preventing the endoplasmic reticulum-mitochondria contact required for mitochondrial fission [100].

In summary, the study of mitochondrial fusion in pancreatic cancer is still at an early stage from a molecular and therapeutic point of view. One of the main challenges is to explain the apparent contradictions found between in vitro and in vivo models. Despi te Li et al. demonstrating that a myoferlin inhibitor has an anti-metastatic activity in pancreatic cancer cells both in vitro and in vivo [101], others showed increased mitochondrial fission found in different PDAC cell lines correlates with the promotion of glycolic flux [81,86]. In contrast, in mice, the higher metastatic capacity of PDAC cells was associated with OXPHOS, increased likely by the expression of proteins involved in mitochondrial fusion [99]. Another approach to evaluate is whether the inhibition of fusion proteins, such as OPA1 and MFN2, has any consequence in the progression and chemoresistance of PDAC, and determine if it could be a realistic therapeutic approach considering their important role also in normal tissues, such as the heart and muscle.

### 3.2. Mitochondrial Fission in Pancreatic Cancer

Since cancer is a disease whose cells show alterations, especially at the proliferative level, studies have been conducted on the relationship between tumor and mitochondrial dynamics in recent decades. In most of these works, it has been described that mitochondrial fission has a pro-tumorigenic role in several cancer types, including lung, colon, breast cancer, melanoma, and hepatocellular carcinoma [25]. Regarding pancreatic cancer, low-moderate levels of DRP1 phosphorylation in Ser-616 have been observed in 11 over 12 pancreatic tumor specimens analyzed. In addition, a high level of Ser616-phosphorylated DRP1 and a fragmented mitochondria phenotype has been found in PDAC cell lines and patient-derived cells [43]. In cellular models with RAS activation (HRas^G12V^ expressing), it has been demonstrated that RAS promotes phosphorylation of DRP1 at Ser616 by direct activation of the MAPK pathway, in particular ERK2, and mitochondrial fragmentation [86]. On the other side, DRP1 knockdown has been shown to inhibit fragmented mitochondria phenotype and tumor cell growth in vitro, in mouse xenograft, and in a genetically engineered mouse model of KRAS-driven pancreatic cancer [85]. A study performed on a cohort of 127 patients showed that DRP1 is significantly more expressed in pancreatic tumor tissue than in adjacent non-tumor tissues. Moreover, Kaplan–Meier analysis reveals that pancreatic cancer patients with high DRP1 expression have a significantly shorter overall survival than those with low DRP1 expression [85]. Furthermore, in addition to its function in mitochondrial dynamics, it has been shown that DRP1 affects the regulation of cell proliferation. Indeed, despite the divergent studies about the link between DRP1 and apoptosis, it has been shown that its knockdown reduces cell growth by inhibiting G1-S cell cycle transition and inducing apoptosis in pancreatic cancer cell lines. Moreover, DRP1 knockdown inhibits pancreatic cancer cell migration and invasion by suppressing matrix metallopeptidase 2; conversely, DRP1 overexpression promotes cell growth, migration, and invasion in PDAC cell lines [85]. The role of DRP1 in pancreatic cancer glycolytic metabolism has also been identified by different authors, who reported some different facets of the role of this protein in energy metabolism regulation. Indeed, Nagdas et al. showed that DRP1 knockdown determines a decrease in Hexokinase II expression and, consequently, in glycolytic flux in both RAS-transformed mouse embryonic fibroblasts and PDAC cells. In addition, they also showed that the loss of DRP1 reduces efficient fatty acid oxidation and electron transport chain functionality, particularly the one of succinate dehydrogenase (SDH) [86]. Liang et al. showed that DRP1 supports aerobic glycolysis, demonstrating that its knockdown dramatically decreased glucose consumption and lactate production. At the same time, its overexpression significantly increased these phenomena in a pancreatic cancer cell line [85].

Interestingly, DRP1 expression can be regulated at the mRNA level by miRNAs; among these, miR-29a, -125a, and -424 have been reported. miR-29a has been identified as a tumor suppressor, and it is downregulated in several different solid tumors (Figure 2). In PDAC tissues, a significantly inverse correlation between the levels of DRP1 and miR-29a was observed. Moreover, a synthetic miR-29a significantly reduces cell growth promoted by DRP1 overexpression in a pancreatic cancer cell line, suggesting that miR-29a downregulation may contribute to the upregulation of DRP1 and, thus, tumor growth and metastasis in pancreatic cancer [85]. Another miRNA implicated in mitochondrial dynamics is miR-125a. The expression level of miR-125a is frequently low in several tumor types, including breast, ovarian, and lung cancer [82]. MiR-125a is significantly less present in the serum of patients with PDAC or other types of digestive-tract cancers in comparison to healthy control individuals. Still, its expression does not show statistical significance in differentiating the cancerous tissue from the normal surrounding tissue (*n* = 10) [102]. In the Panc1 cell line, miR-125a is present at low levels and its reintroduction causes mitochondria fission via MFN2 downregulation. MiR-125a overexpression promotes mitochondrial cell death by inducing mitochondrial membrane dissipation, cytochrome c leakage, and caspase activation. Furthermore, a mimic miR-125a reduces electron transport chain activity, glucose consumption, and lactate production [82]. Also, miR-424 regulates mitochondrial dynamics. Indeed, the miR-424 level is significantly higher in pancreatic tumor tissues than in peritumoral tissues. A correlation between the mRNA expression levels of this miRNA and MiD49, which is a DRP1’s receptor expressed in the outer membrane of mitochondria, has been shown in tumor tissues [87]. The relation of miR-424 with MiD49 supports the evidence that the role of mitochondrial dynamics in the sustainment of tumor proliferation and malignancy is not univocal in favor of fusion or fission and that more studies should be performed to understand the real functions of DRP1 receptors. Indeed, a synthetic precursor of miR-424 has significantly decreased MiD49 mRNA and protein levels in pancreatic cancer cell lines. In addition, MiD49 expression is significantly lower in the pancreatic tumor tissues compared to the adjacent non-tumor tissues, and PDAC patients with a low expression level of MiD49 have clearly poorer overall survival than those with high MiD49. Additionally, in pancreatic cancer cell lines, MiD49 is downregulated, and its overexpression determines a marked mitochondria fragmentation, ROS levels increase, G1-S phase cell cycle arrest, and cell apoptosis. Moreover, MiD49 overexpression reduces the migration and invasion of PDAC cell lines by inhibiting epithelial-mesenchymal transition (Figure 2). Finally, MiD49 overexpression suppresses PDAC growth and metastasis both in vitro and in vivo [87]. In addition to this study, it has been demonstrated that mitochondrial Ca^2+^ signaling is related to mitochondrial fission through the regulation of DRP1. The expression level of SMDT1, a subunit of mitochondrial calcium uniporter (MCU) complex, is significantly lower in PDAC cancers than in paired adjacent tissues, significantly correlating with PDAC prognosis. SMDT1 overexpression significantly decreases proliferation rates of PDAC cell lines and induces mitochondria-mediated apoptosis with cytochrome c release and caspase activation. Moreover, SMDT1 overexpression significantly increases the number of fragmented mitochondria with a decrease in elongated, reticular, and intermediate mitochondria, increasing significantly Ser616-phosphorylated DRP1 and FIS1 (Figure 2) [88]. Discrepancies found in the effect of mitochondrial fission on tumor cell growth could be attributable to the non-direct effect of DRP1 or to the extent of mitochondrial fragmentation, and, certainly, the role of mitochondrial fission as a pro-oncogene needs further investigation.

Altogether, considering all the studies about the role of mitochondria dynamics in PDAC that have been performed on different cellular systems (including cell lines, animal models, and patients), we can generally refer to a preference for fission processes in the sustainment of proliferation and migration. In addition, the use of fission inhibitors has been proposed by some authors as a new potential strategy to reduce tumor growth and increase chemotherapy sensitivity. However, many studies are still necessary, hopefully not only on cell lines but also on animal models or patients’ derived tissues, to characterize the role of mitochondrial dynamics in PDAC.

## 4. Mitochondria Dynamics in Pancreatic Cancer Stem Cells

As already deeply disclosed above, pancreatic cancer is one of the most malignant tumors. The elements that determine such high cancer aggressiveness are different: from a clinical point of view, the lack of early diagnostic markers and high resistance to standard therapies, while from a biological point of view, the strong tendency to form metastasis and relapse. All these features can be mainly attributed to the presence of a subpopulation of cells known as cancer stem cells (CSCs). Even if they represent only 0.1–1% of the bulk of the tumor [103], in recent years, an increasing number of studies have shown how much these cells play a critical role in cancer and are responsible for its aggressiveness, chemoresistance, and metastatic potential. A peculiar feature of CSCs is their link with the epithelial-to-mesenchymal transition (EMT) that strictly associates them with the dissemination and formation of metastasis. It has been hypothesized that there are different degrees of induction of EMT. When there is a strong activation, the achievement of a complete mesenchymal phenotype, cells could intravasate, migrate throughout the bloodstream, and extravasate, then colonize a secondary organ. To do this, cells must undergo the opposite mechanism, which is the mesenchymal-to-epithelial transition (MET) [104]. EMT activation is necessary not only for the physical dissemination of cancer cells, but it has been reported that it could be necessary also for their entrance into the CSC state. However, the mechanism is still unknown since it is very difficult to demonstrate. Recently, we have shown that during progressive de-differentiation CSCs also undergo, among the modifications cited above, a metabolic change and shift from a more glycolytic to a more oxidative metabolism [105]. This remodeling may be linked to mitochondria dynamics and could be important in determining the cell state. Indeed, given the striking changes in mitochondrial architecture that occur when stem cells differentiate and are reprogrammed, it is reasonable to ask what role mitochondrial dynamics might play [58]. Mitochondrial dynamics are essential for successful asymmetrical division in normal stem cells [106]. They have been linked to the proliferation and survival of stem cells in normal tissues and some cancer types [58,107]. Thus, as pancreatic CSCs are particularly dependent on the activity of their mitochondria, it might be important to focus on mitochondrial dynamics as a critical process in the homeostasis of these organelles. As reported above, important notions emerged in the description of the delicate balance between mitochondrial fission and fusion in cancer [108], especially in PDAC. However, the attempts to rich the comprehension of these events are difficult to pursue, probably because we have to consider the presence of CSCs, which have some key features, including the plasticity of their metabolism, that may render it difficult to delineate their mitochondrial assessment. Although the exiguity of information on mitochondrial regulation in CSCs, particularly in pancreatic CSCs, renders it difficult to delineate their mitochondrial dynamic settings, some preliminary aspects have been highlighted by some authors. Indeed, due to their intrinsic aggressiveness, cancer stem cells represent an essential target for designing effective treatments against pancreatic cancer. Previous data indicate that it is necessary to consider that the steps of tumor and metastasis development pass through a metabolic plasticity of cancer cells that has to be further characterized to develop the best strategy for an effective cancer eradication [105]. As recently described, pancreatic CSCs also rely on mitochondrial metabolism to maintain their stemness, representing a putative target for their elimination [108]. In this regard, Sancho et al. discovered that pancreatic CSCs are particularly sensitive to mitochondrial targeting due to their dependence on OXPHOS [109]. According to this, it has been demonstrated that perturbing mitochondrial function by either inhibiting the electron transport chain or altering its redox state. This leads to significantly decreased pancreatic CSCs functionality and chemoresistance [107,110]. Thus, these results identified mitochondrial activity as a key vulnerability for pancreatic CSCs. Nevertheless, the relationship between mitochondrial dynamics and stemness in this cancer type has not yet been well understood. Indeed, Courtois et al. highlighted that pancreatic CSCs are characterized by an accumulation of small mitochondria and an increased DRP1/MFN2 ratio compared to the differentiated counterpart, indicating that these cells rely on mitochondrial fission. This event is described as mostly confined to the CSC subpopulation. They also demonstrated that in PDAC tissues, DRP1 is overexpressed, and this signature is related to lower survival in patients with pancreatic cancer. Moreover, the pharmacological inhibition of fission results in the accumulation of dysfunctional mitochondria that leads to two different aspects: on the one hand, an energy crisis with consequent cell death for apoptosis; on the other hand, the inhibition of stemness-related tumorigenicity and invasiveness, and chemosensitization to gemcitabine, one of the most used drugs for the treatment of pancreatic cancer. This gave hope to a promising target to fight the tumor, also because it diminished CSC content in PDAC patient-derived xenografts. However, there are still profound contradictions, especially concerning CSC metabolism, which has been described to be based on either OXPHOS or glycolysis or to be plastic, and mitochondrial dynamics, defined in favor of elevated fission activity that results in mitochondria fragmentation and impaired oxidative phosphorylation. This discrepancy highlights the strong need for further studies and in-depth analyses on this complex and important topic. Many questions still need to be answered, and further investigation will serve to shed light on all of this.

## 5. Concluding Remarks

Pancreatic ductal adenocarcinoma is a highly lethal neoplasia, and the currently used therapeutic approaches are not effective in a wide range of patients. Indeed, PDAC is one of the oncologic diseases with the lowest 5-years survival rate. As reported above, its aggressiveness is due to the absence of consistent early diagnostic markers and efficacious therapies. Thus, there is a need to deeply study the biological features of pancreatic cancer and cancer stem cells to improve patient survival. Due to the importance of regulating cellular functions, especially metabolic energy, mitochondria may represent key targets to be hit. However, as deeply described in this review, these organelles are plastic, and their fusion and division process represents an aspect that could discriminate among normal, tumoral, and cancer stem cells. In fact, modification of the fusion/fission equilibrium within the cells affects cellular function, both in physiological and pathological conditions. Despite the increasing interest in this aspect, several authors have investigated mitochondrial dynamics in cancer cells reporting controversial results that do not clarify whether cancer cells possess more fused or elongated mitochondria. However, from a deep analysis of the data present in the literature, it appears that fusion/fission balance may be different according to the tumor type, without forgetting the intratumoral variability that has been widely described for almost all the neoplasia (Table 1). Concerning pancreatic cancer, even if these discrepancies are reported in the literature [111], we can generally point out that most studies report that PDAC cells preferentially show fragmented mitochondria. Indeed, different studies reporting functional assays on key proteins regulating fusion and fission, including OPA-1 and DRP1, highlight that overall fission has a crucial role in the maintenance of tumor proliferative capacity, migration potential, generation of ROS together with increased genomic instability, a decrease of patient survival, and reduction of metastatic potential of the cells as demonstrated in cell lines, animal models, tumor tissues, and patients. In addition, due to mitochondria’s metabolic role, the metabolism has been taken into account in many studies on PDAC, with a general assumption that altered mitochondria fission in cancer cells causes a disbalance of the glycolytic/oxidative phosphorylation metabolism, with consequences on cell proliferation and aggressiveness. However, due to the difficulties and high variability in studying metabolism in vitro, it remains necessary to comprehend the molecular mechanisms through which the OXPHOS and glycolysis are regulated by fission in PDAC.

Finally, the use of fission inhibitors, such as mdivi-1 and Drpitor1, further sustains the preference of cancer cells to maintain fragmented mitochondria to preserve their proliferative capacity, supporting that inhibition of mitochondrial fission could be a prospective strategy to reduce tumor growth and increase chemotherapy sensitivity. However, to delineate a potential therapeutic strategy based on mitochondrial dynamics in PDAC, it is necessary to perform studies on conditional knockout mouse models of key fission/fusion proteins and tumor tissue specimens in comparison to peri-tumoral normal tissue from the same patients.

In conclusion, it is still necessary to perform more studies to characterize the role of mitochondrial dynamics in PDAC, considering that it may be a key target for the generation of new promising therapies and that the exploitation of these therapeutic approaches should necessarily avoid secondary effect on normal tissues, where mitochondria dynamics physiologically take place.

## Figures and Tables

**Table 1:** Drugs and/or methods that target mitochondrial fusion and fission in cancer.

**Mitochondrial Fusion**					
**Tumor Type**	**Players**	**Functional Effect**	**Method**	**Results**	**Reference**
PDAC	OPA1	Pro-tumor	Inhibition of the interaction between Hsp90 and OPA1 (loss of myoferlin)	Reduction in mitochondrial cristae amount, energy production, cell proliferation, and induction of autophagy	Rademaker et al., 2018 [81]
PDAC	MFN2	Anti-tumor	Overexpression of *MFN2*	Improvement of survival in preclinical models, by the promotion of autophagy and the reduction in mitochondrial mass, OCR, and ATP production	Yu et al., 2019 [75]
PDAC	fusion	Anti-tumor	Pharmacological induction of mitochondrial fusion by leflunomide	Improvement of survival in mouse models	Yu et al., 2019 [75]
PDAC	MFN2	Anti-tumor	Downregulation of MFN2 by miR125-a	Increased fission as a tumor suppressor process	Pan et al., 2018 [82]
PDAC	UCA1	Pro-tumor	UCA1 knockdown	Decreased cell viability and induced apoptosis and mitochondria fragmentation	Teng et al., 2021 [83]
PDAC	Myoferlin	Pro-tumor	Decreased levels of Myoferlin	Reduced cell proliferation and induced aoutophagy	Rademaker et al., 2018 [81]
Liver cancer	OPA1 - MFN1	Pro-tumor	Knockdown of *OPA1* or *MFN1*	Inhibition of the tumor formation in vivo in mice	Li et al., 2020 [77]
Several cancer types	OPA1	Pro-tumor	Deletion of endothelial *OPA1*	Decrease of tumor angiogenesis, growth, and metastasis	Herkenne et al., 2020 [79]
Several cancer types	OPA1	Pro-tumor	Inhibition of OPA1 by MYLS22	Decrease of tumor angiogenesis, growth, and metastasis	Herkenne and Scorrano, 2020 [84]
Several cancer types	MFN2	Pro-tumor	Deletion of *MFN2*	Reduction of cell proliferation	Yao et al., 2019 [78]
**Mitochondrial Fission**					
**Tumor Type**	**Players**	**Functional Effect**	**Method**	**Results**	**Reference**
Pancreatic cancer/PDAC	DRP1	Pro-tumor	DRP1 knockdown	Inhibition of fragmented mitochondria phenotype, and tumor cell growth in vitro and in mouse xenograft	Liang et al., 2020 [85]
Pancreatic cancer/PDAC	DRP1	Pro-tumor	Inhibition of DRP1 by synthetic miR-29a	Reduction of cell growth in vitro	Liang et al., 2020 [85]
PDAC	DRP1	Pro-tumor	DRP1 knockdown	Decrease in Hexokinase II expression and glycolytic flux	Nagdas et al., 2019 [86]
PDAC	MiD49	Anti-tumor	Overexpression of MiD49	Suppression of PDAC growth and metastasis both in vitro and in vivo	Bai et al., 2020 [87]
PDAC	SMDT1	Anti-tumor	SMDT1 overexpression	Decrease of proliferation rates of PDAC cell lines	Xie et al., 2019 [88]
Breast cancer	DRP1	Pro-tumor	Silencing of DRP1	Reduction of cell migration and invasion	Zhao et al., 2013 [72]
Breast and lung cancer	DRP1	Pro-tumor	Inhibition of DRP1 by Drpitor1	Damage of oxidative metabolism and induction of cell death	Wu et al., 2020 [74]
Several cancer types	DRP1	Pro-tumor	Inhibition of DRP1 by mdivi-1	Damage of oxidative metabolism and induction of cell death	Dai et al., 2020 [73]
